# Monitoring Two Typical Marine Zooplankton Species Using Acoustic Methods in the South China Sea

**DOI:** 10.3390/s24154827

**Published:** 2024-07-25

**Authors:** Jing Liu, Yong Tang

**Affiliations:** 1 School of Fishery, Zhejiang Ocean University, Zhoushan 316022, China; 2 College of Marine Living Resource Science and Management, Shanghai Ocean University, Shanghai 201306, China; y-tang@shou.edu.cn

**Keywords:** zooplankton outbreak, broadband echosounder, volume scattering strength, density

## Abstract

The broadband scientific echosounder is considered to have great potential for zooplankton monitoring. In this study, two common types of zooplankton in the South China Sea, *Rhopilema hispidum* and *Acetes chinensis*, were continuously monitored using a broadband scientific echosounder. The results revealed distinct volume scattering strength (SV) spectral characteristics between the echoes of *R. hispidum* and *A. chinensis*. Meanwhile, echoes of *R. hispidum* and *A. chinensis* were classified using the k-means clustering algorithm, achieving an 83.4% accuracy rate. The SV value at a nominal frequency of *R. hispidum* changes more sharply than that of *A. chinensis*, suggesting that the density of *R. hispidum* changes more dramatically. This study demonstrates the advantages of monitoring *R. hispidum* and *A. chinensis* outbreaks with a broadband scientific echosounder.

## 1. Introduction

*Acetes chinensis* and *Rhopilema hispidum* are two kinds of macro- and mega-zooplankton frequently having emerged in large numbers in the South China Sea in recent years. *A. chinensis* is a species of macroplankton shrimp belonging to the family Sergestidae, which inhabits the coastal areas of the Indo-West Pacific, including China, Korea, and Japan [1]. Despite the small carapace length of the shrimps ranging from around 3 mm to 11 mm in the Yellow Sea [2], *A. chinensis* is one of the most important fishery resources in China due to its large biomass [3]. *R. hispidum* is a species of jellyfish belonging to the genus Rhizostomidae, with an umbrella diameter ranging from 350 to 540 mm. Cases of *R. hispidum* outbreaks have been discovered off the coasts of China’s Hainan and Guangdong provinces in recent years. *R. hispidum* is a commercially important fishery resource [4]. Therefore, it is economically and ecologically indispensable to detect the distributions of these two species and estimate their biomasses in the South China Sea.

Zooplankton is typically collected and monitored using a fine-mesh net [5]. However, this method of net sampling is still subject to some limitations. For instance, the net sampling method is restricted to a limited depth range and cannot easily adapt to varying depths. The process of implementation and biological collection is labor-intensive. A balance must be struck between mesh size and the targeted organisms. Achieving consistent, long-term monitoring presents its own set of challenges. For these reasons, there is a desire for fast, stable, and economical methods to monitor zooplankton, replacing traditional network sampling methods. Acoustic methods utilize underwater sensors (e.g., echosounders) to capture the abundance and distribution of aquatic organisms, enabling the rapid acquisition of large amounts of high-resolution data. Studies of zooplankton monitoring by acoustic methods have emerged in recent years [6,7,8,9,10]. Echosounders can transmit sound pulses into the water and receive echoes from scatterers such as fish and the seabed. By dividing the volume scattering strength (SV) by the average target strength (TS) of the target organism, density information can be obtained [11], which is the basic principle for assessing biomass using acoustic methods. The TS of organisms is related to their species, size, and biological characteristics of organisms. Therefore, one of the challenges in using acoustic methods to monitor underwater organisms is identifying the species based on their echo characteristics. The acoustic classification of nekton species such as fish has been conducted frequently [12,13,14,15]. Acoustical observation and recognition using narrowband and broadband echosounders have also been attempted on zooplankton species [6,16,17,18,19,20,21]. Broadband echosounders are more advantageous for plankton monitoring compared to traditional narrowband echosounders. Broadband echosounders can transmit linear frequency-modulated (LFM) signals. Echoes with a high range resolution and a high signal-to-noise ratio (SNR) can be obtained after pulse compression processing. Furthermore, the echoes collected using broadband echosounders also contain spectral information, enhancing the capability for acoustically classifying underwater organisms [20,22].

This study will focus on the two common zooplankton species that have frequently triggered zooplankton outbreaks in the South China Sea in recent years. The aim is to explore the viability of employing a broadband echosounder as an acoustic sensor for monitoring and classifying zooplankton outbreaks. The research will offer technical support for long-term monitoring and an early warning system for zooplankton outbreaks. Furthermore, it is expected to provide technical support in exploring the relationship between zooplankton outbreaks and marine environmental pollution in the future.

The rest of this article is organized as follows: The second part introduces the materials and methods of this study, including biological sampling, acoustic monitoring methods, acoustic dataset collection, and acoustic data processing. The third part presents the results, including the SV spectral characteristics of organisms *R. hispidum* and *A. chinensis*, acoustic characteristic variables, and the variation of SV over time. The fourth part discusses the broadband acoustic scattering characteristics and acoustic classification of *R. hispidum* and *A. chinensis*.

## 2. Materials and Methods

### 2.1. Data Collection

The experiment site is in the waters of Yamen Estuary, to the west of Dajin Island in the South China Sea. This location was selected based on information from previous studies with fishermen, indicating recent outbreaks of jellyfish and *A. chinensis* in the area. The experiments were conducted from 9:00 to 24:00 on 6 April 2023, and from 0:00 to 24:00 on 18 January 2024. These two dates were selected based on conversations with fishermen who reported increased occurrences of *R. hispidum* and *A. chinensis* outbreaks during the chosen season.

In this study, a Simrad EK80 broadband echosounder (Kongsberg Maritime, Horten, Norway) was used to collect acoustic data. The EK80 system mainly consists of a wide-band transceiver Mini and split-beam broadband transducers (ES70-7C). The EK80 system was mounted on a buoy, and the transducer was fixed 2 m below the water surface. Considering the survey area’s water depth of around 7 m, the transducer was set at a horizontal downward angle of about 7 degrees to expand the observation volume. Additionally, the transducer beam direction was ensured to be perpendicular to the water current direction. The broadband echosounder system was calibrated with a 38.1 mm dia. standard sphere made of tungsten carbide with 6% cobalt binder (WC) according to the standard method of Simrad EK80 software (ver.2.0.0) before and after the experiment [23]. Table 1 presents the specifications and settings of the Ek80 system used in this experiment.

Biological sampling was conducted using a set net located approximately 10 m away from the EK80 system. The mouth of the set net was positioned against the water current, and the mesh size of the bag section of the set net was 5 mm × 5 mm to effectively capture organisms such as jellyfish *R. hispidum* and small shrimp *A. chinensis*. Upon the completion of data acquisition using the EK80 system, the organisms captured in the set net were retrieved, and their species were identified onshore. Subsequently, the collected organisms were weighed, and the biological sampling data were utilized to validate the types of echoes detected.

### 2.2. Process of Acoustic Data

The raw data recorded using the EK80 system were processed using Echoview (ver.13.0, Echoview Software Pty Ltd., Hobart, Australia), a specialized fishery acoustic data processing software. When there are a large number of biological targets in the sampled volume, their echoes will form a received signal, making it impossible to distinguish the individual targets. In this case, the SV can be used to reflect the total biomass of the targets in the sampled volume [11]. The SV spectra indicate that SV varies as a function of acoustic frequency. The SV spectra ranging from 55 to 90 kHz were analyzed to classify species of *A. chinensis* and *R. hispidum*. When calculating SV spectra, the Ping number and sampling window used for fast Fourier transform (FFT) need to be specified first. The SV spectra were calculated using FFT and the sonar equation, based on the recorded voltage data within the sampling window [24]. In this study, for every ping datum, the window size of the sampling window was set at 1 m. Data above 5 m and below 15 m from the transducer surface were excluded from the analysis to avoid interference from the water surface and seabed. To ensure the stability of the calculated SV spectra, the process involved averaging the SV over every 100 pings of data [22]. Such calculations were repeatedly performed throughout the recorded time period, and the resulting time series was documented. The recorded data with average SV values below −75 dB at 70 kHz were disregarded for lacking sufficient biological signal. Henceforth, unless noted, the term “SV spectra” denotes the average calculated from every 100 pings.

The shape and amplitude of the SV spectra are influenced by factors such as species, density, biological size, and tilt angle. Therefore, the typical SV spectra of different biological echoes are considered to be different. Therefore, species and biological information can be distinguished based on the spectral characteristics of SV spectra from different organisms [20,22]. To distinguish between *A. chinensis* and *R. hispidum*, their SV spectra were further analyzed, and the following five characteristic variables were extracted from each SV spectrum: I. The SV value at nominal frequency [$S_{v}$(70 kHz)], which represents the echo strength at 70 kHz. In the case of a specific TS, a higher value of $S_{v}$(70 kHz) corresponds to a higher biological density; II. The variance (*σ*) of the SV value among different frequencies, which represents the stability of the SV value across various frequencies; III. Correlation coefficient ($R^{2}$) for linear fitting of SV spectra. The closer the $R^{2}$ value is to 1, the better the linear fit of the SV spectrum; IV. The increment of SV in bandwidth (${∆S}_{vband}$), ${∆S}_{vband}$ = *a* × (90-55), where *a* means the slope in the linear fitting of the SV spectrum and the (90-55) represents the bandwidth of the signal transmitted by the transducer, V. The curvature (*k*) of the SV spectrum, the *k*, can be calculated using the three-point [$S_{v}$(60 kHz), $S_{v}$(72.5 kHz), $S_{v}$(85 kHz)] method [25]. The curvature is a measure that describes the degree of curvature of the SV spectrum, and the greater the *k*, the greater the degree of the curvature of the SV spectrum curve. These variables were hereby used as parameters to classify *A. chinensis* and *R. hispidum* using an unsupervised classification method (K-means). Before performing K-means clustering, the characteristic variable dataset mentioned above was first standardized to eliminate the influence of variable scales and dimensions. In this study, the “kmeans” function from the “stats” package of R software (version 4.3.3) was utilized to conduct the clustering analysis.

The $S_{v}$(70 kHz) was used to evaluate the biomass change over time of *A. chinensis* and *R. hispidum* because $S_{v}$ = $n\cdot T_{s}$ [11], where $S_{v}$ is the linear value of SV, $T_{s}$ is the linear value of TS, and *n* is the density of the organisms. Although there currently is not enough knowledge of TS concerning *A. chinensis* and *R. hispidum*, $S_{v}$(70 kHz) can also indicate the relative values of density and biomass.

## 3. Results

On 6 April 2023, the catch from the set net totaled 760 kg, comprising 10 kg of small fish and 750 kg of jellyfish. Among them, the small fish mainly consisted of *Ambassis gymnocephalus* with body lengths of less than 6 cm, while the jellyfish were predominantly large jellyfish *R. hispidum* with umbrella diameters of less than 80 cm. No *A. chinensis* was observed in the catch. Approximately 98.7% of the catch’s weight consisted of *R. hispidum*, indicating that the echoes recorded on 6 April 2023, were predominantly from this species. The catch from the set net on 18 January 2024, totaled 370 kg, comprising 24 kg of small fish (mainly A. *gymnocephalus* with body lengths of less than 6 cm), 346 kg of *A. chinensis*, and a few small jellyfish with umbrella diameters of less than 2 cm. *A. chinensis* accounts for approximately 81.4% of the catch; therefore, the echoes on 6 April 2023, are primarily attributed to *A. chinensis*.

**Figure 1:**
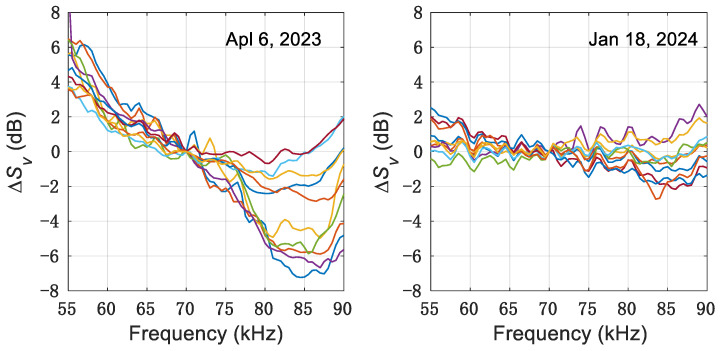
Relative frequency response of some typical SV spectra. Left: ∆SV spectra from *R. hispidum* on 6 April 2023; right: ∆SV spectra from *A. chinensis* on 18 January 2024.

A total of 260 and 350 SV spectra were obtained on 6 April 2023, and 18 January 2024, respectively. Due to variations in organism density, the amplitudes of SV spectra differed. For ease of representation, Figure 1 shows the relative frequency response (∆SV spectra) of some typical SV spectra of *A. chinensis* and *R. hispidum*. $∆S_{v}\left(f\right)=S_{v}\left(f\right)-S_{v}\left(70\;kHz\right)$, where $∆S_{v}\left(f\right)$ is the ∆SV spectra [26]. The results indicate different shapes of the SV spectra. The SV values for *R. hispidum* exhibited a decline with rising frequency, in contrast to *A. chinensis*, whose SV values showed little variation. This disparity highlighted a clear distinction in the spectral profiles of SV for the two organisms.

**Figure 2:**
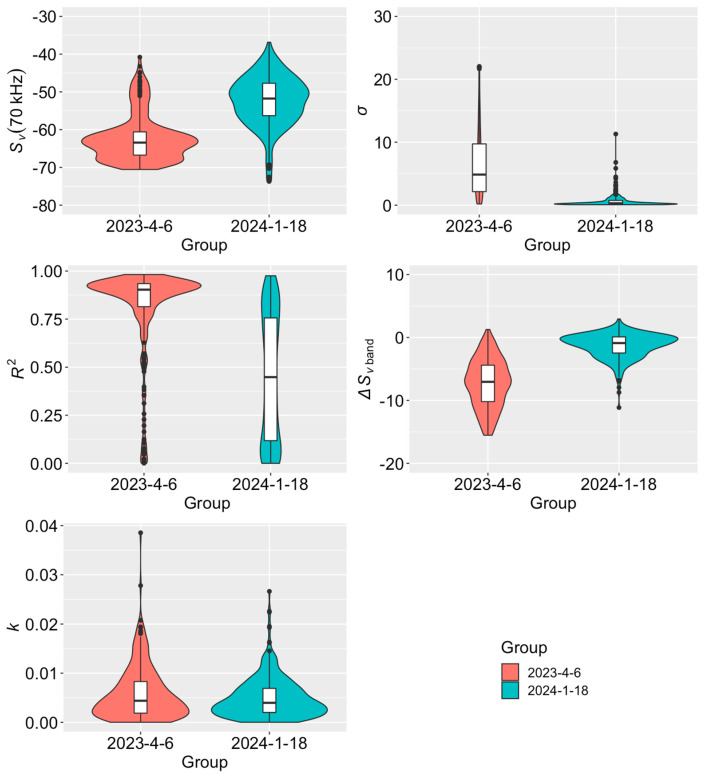
Violin plots of $S_{v}(70\;kHz)$, σ, $R^{2}$, ${∆S}_{vband}$, and k from the SV spectra of *R. hispidum* and *A. chinensis* observed on 6 April 2023 and 18 January 2024.

Figure 2 shows the five characteristic variables [$S_{v}(70\;kHz)$, σ, $R^{2}$, ${∆S}_{vband}$, and *k*] from the SV spectra of *R. hispidum* and *A. chinensis* observed on 6 April 2023 and 18 January 2024. It could be observed that the distribution of the four variables, except for *k*, varied significantly between the two groups of organisms. The *p*-values of Student’s *t*-test (with a significance level set at 0.01) were $1.91\times 10^{-61}$, $1.30\times 10^{-45}$, $2.27\times 10^{-49}$, $9.04\times 10^{-73}$, and 0.02, respectively. The $S_{v}(70\;kHz)$ values of *R. hispidum* are mostly distributed between −70 and −58 dB, while the $S_{v}(70\;kHz)$ values of *A. chinensis* are mostly distributed between −65 and −38 dB. This is due to the density of *A. chinensis* being much higher than that of *R. hispidum*. In most cases, the *σ* values of *R. hispidum* are larger than those of *A. chinensis*, indicating that the SV value of *R. hispidum* varies greatly between different frequencies. The $R^{2}$ scores of *R. hispidum* are mostly above 0.75, while the $R^{2}$ values of *A. chinensis* are discretely between 0 and 1. This indicates that the SV spectra of *R. hispidum* have a high degree of linear fitting in most cases, while the SV values among frequencies of *A. chinensis* have a relatively large degree of variation in distribution, sometimes resulting in a low degree of linear fitting. The ${∆S}_{vband}$ values of *R. hispidum* are mostly less than 0, while the ${∆S}_{vband}$ values of *A. chinensis* are distributed around 0, indicating that the SV spectra of *R. hispidum* mostly show a decreasing trend with frequency, consistent with the results in Figure 1. The *k*-value distributions of *R. hispidum* and *A. chinensis* are similar, suggesting that the curvature of their SV spectra curves is alike. The above results indicate that there is significant specificity in the SV spectra of *R. hispidum* and *A. chinensis*, and acoustic classification based on the SV spectrum characteristics of both species is feasible.

**Figure 3:**
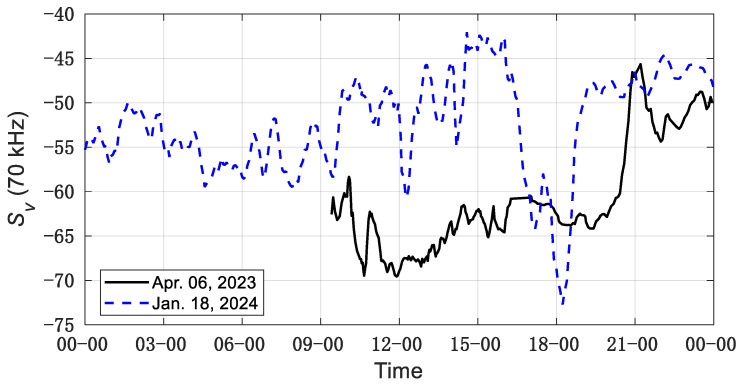
The variation in the SV values at the nominal frequency over time. The black line represents the data for *R. hispidum* observed from 9:00 to 24:00 on 6 April 2023, and the blue dotted line represents the data for *A. chinensis* observed from 0:00 to 24:00 on 18 January 2024.

Figure 3 shows the variation in SV values at nominal frequency over time, indicating the dynamic fluctuations in density and biomass of *R. hispidum* and *A. chinensis*. As shown in the figure, the $S_{v}(70\;kHz)$ of *R. hispidum* increased since 6 p.m., indicating a rise in the density of *R. hispidum* during the night. In contrast, the $S_{v}(70\;kHz)$ value of *A. chinensis* remained relatively stable over the 24 h period, except for a brief sudden drop at 6 p.m. Although the current data cannot explain the cause of the sudden change in *R. hispidum* density, the above results indicate that environmental factors such as time and light intensity can affect the distribution of zooplankton.

## 4. Discussion

Most research on the acoustic scattering of jellyfish has been conducted in experimental tanks. Moreover, there appears to be a gap in the literature regarding the broadband acoustic scattering properties of *A. chinensis*. In this study, the continuous broadband echoes of *R. hispidum* and *A. chinensis* were successfully observed using a broadband scientific echosounder. The SV spectra of the two organisms were analyzed, and the characteristics of the SV spectra were extracted. This analysis has shown distinct SV spectra for the two zooplankton species, proving the feasibility of acoustic classification. It highlights the benefits of acoustic methods for long-term monitoring of zooplankton outbreaks and for exploring their connection to marine environmental pollution.

Herein, the ∆SV spectra of *R. hispidum* showed an obvious decreasing trend with increasing frequency (see Figure 1), yet the slope of the ∆SV varied. Compared to *A. chinensis*, the ∆SV spectra of *R. hispidum* exhibited greater variability, indicating that the TS of *R. hispidum* also had greater variability, and further implying the need for caution when predicting the biomass of *R. hispidum*. Indeed, the variation in TS could lead to significant differences in biomass estimation. In this study, the ∆SV spectra of *R. hispidum* at 55–90 kHz exhibited a similar trend to that of *Chrysaora chesapeakei* in Rachel’s study [27]. The instability of these spectra shapes might be caused by differences in the material characteristics of jellyfish, such as sound velocity ratio (biological tissue sound speed and water sound speed) and density ratio (biological tissue density and water density). Furthermore, the size and growth stage of jellyfish were also significant contributors to this instability. The ∆SV spectra of *A. chinensis* were more stable compared to *R. hispidum* (see Figure 1 and the σ in Figure 2), possibly due to the stable biological characteristics of *A. chinensis*, such as the density ratio, sound speed ratio, and size. Meanwhile, the ∆SV spectra slope of *A. chinensis* exhibited both positive and negative values, which might be attributed to two reasons. Firstly, *A. chinensis* exhibited different behaviors across various time periods, leading to variations in tilt angle distribution and, consequently, differences in ∆SV spectra. Secondly, the SV spectra of *A. chinensis* could be undergoing a transition from Rayleigh scattering to geometric scattering [20,22]. In general, the slope of *A. chinensis* was smaller than that of *R. hispidum* (see Figure 1). This fully demonstrated the specificity of the SV spectra of the two organisms and the feasibility of classifying the two organisms using acoustic methods.

**Figure 4:**
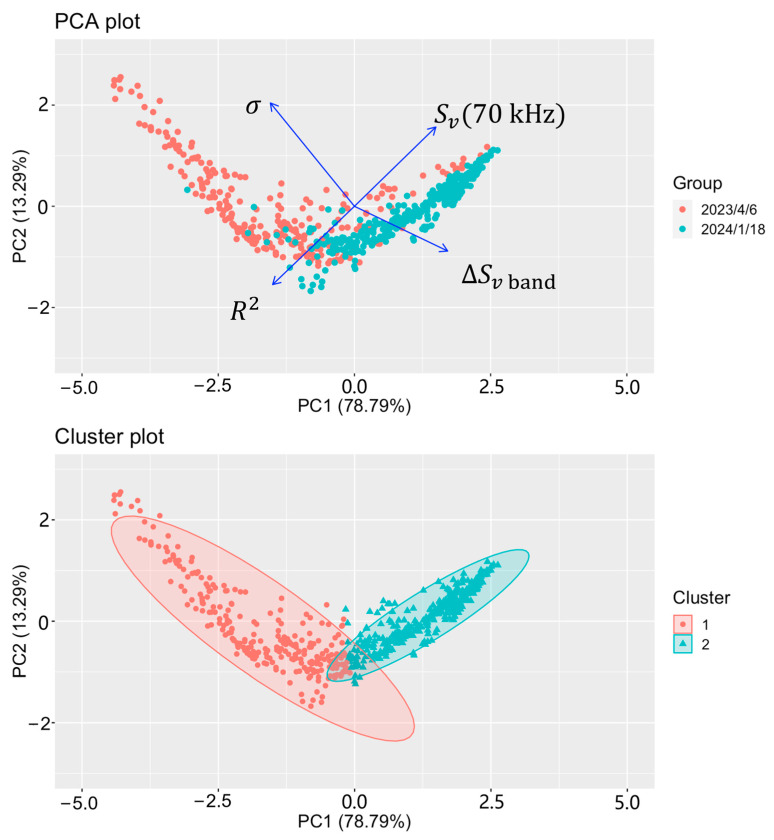
Principal Component Analysis (PCA) and k-means clustering based on SV spectrum characteristics of *R. hispidum* and *A. chinensis* observed on 6 April 2023 and 18 January 2024, with the ellipses representing the confidence intervals.

In this study, five characteristic variables were extracted from the SV spectra to quantify the differences between the SV spectra of *R. hispidum* and *A. chinensis*. These variables not only contained information regarding the scattering strength of biological echoes but also data concerning the shape of the SV spectra. The $S_{v}(70\;kHz)$ of *R. hispidum* was concentrated between −70 and −57 dB (see Figure 2). The range from −57 dB to −40 dB was thought to be attributed to a small number of strong echoes from the school of fish. The $S_{v}(70\;kHz)$ of *A. chinensis* was concentrated between −65 and −40 dB (see Figure 2). This is because the density of *A. chinensis* was much higher than that of *R. hispidum*. From the four variables, in Figure 2, excluding *k*, it could be found that the SV spectra of the two zooplankton exhibited different spectral characteristics. Therefore, the Principal Component Analysis (PCA) algorithm was further employed to process the SV spectrum characteristic parameters of *R. hispidum* and *A. chinensis* obtained on 6 April 2023, and 18 January 2024. Subsequently, cluster analysis was performed on these SV spectra using the k-means algorithm, and the results are shown in Figure 4. The k-means clustering algorithm achieved an 83.4% accuracy rate in correctly classifying *R. hispidum* and *A. chinensis*, demonstrating a strong classification effect. Misclassified samples might be attributed to echoes from other organisms, such as A. *gymnocephalus*. In previous studies, characteristic parameters such as the shape of fish school echo and geographical environment information have also been used for acoustic species classification [28,29,30]. In the future, these parameters could be potentially included to enhance the accuracy of *R. hispidum* and *A. chinensis* classification.

In this study, the presence/absence of *R. hispidum* and *A. chinensis* was monitored using $S_{v}(70\;kHz)$, demonstrating the feasibility of employing acoustic methods for monitoring the presence/absence of *R. hispidum* and *A. chinensis*. However, due to the lack of TS information for *R. hispidum* and *A. chinensis*, the conversion of echo intensity to biomass for these two organisms was hereby not included. In order to achieve acoustic biomass monitoring and early warning in the future, it is essential to clarify the TS of *R. hispidum* and *A. chinensis*. In the future, the TS of *R. hispidum* and *A. chinensis* could also be measured and predicted using laboratory measurements and theoretical models, such as the Distorted Wave Born Approximation (DWBA) model [27,31].

## 5. Conclusions

The findings in the current study offer valuable insights that can contribute to the development of zooplankton monitoring systems with acoustic methods. This study demonstrated the distinct SV spectra characteristics from different zooplankton species, proving the feasibility of monitoring and remote classification of macro- and mega-zooplankton using a broadband echosounder. In the future, the long-term monitoring of zooplankton resources using broadband echosounders and exploring their relationships to the marine environment will contribute to the management of marine resources and their sustainable use through fisheries.

## Figures and Tables

**Table 1:** Specification and settings of the broadband echosounder used in this study.

Settings	Values
Transducer type	ES70-70
Nominal frequency (kHz)	70
Pulse duration (ms)	1.024
Transmit electric power (W)	525
Transmit signal type	LFM
Transmit frequency range (kHz)	55–90
Ramping	Fast

## Data Availability

The raw data are available upon reasonable request to the corresponding author.
